# Genetic Connectivity among Populations of an Endangered Snake Species from Southeastern Australia (*Hoplocephalus bungaroides*, Elapidae)

**DOI:** 10.1002/ece3.25

**Published:** 2011-10

**Authors:** Sylvain Dubey, Joanna Sumner, David A Pike, J Scott Keogh, Jonathan K Webb, Richard Shine

**Affiliations:** 1 School of Biological Sciences, University of SydneyNSW 2006 Australia; 2 Museum Victoria11 Nicholson St, Carlton Gardens, VIC 3053, Australia; 3 Present address: School of Marine and Tropical Biology, James Cook UniversityAustralia; 4 Research School of Biology, The Australian National UniversityCanberra, ACT 0200, Australia

**Keywords:** Australia, conservation genetics, parentage analyses, reptile, Elapidae, microsatellite loci

## Abstract

For endangered species that persist as apparently isolated populations within a previously more extensive range, the degree of genetic exchange between those populations is critical to conservation and management. A lack of gene flow can exacerbate impacts of threatening processes and delay or prevent colonization of sites after local extirpation. The broad-headed snake, *Hoplocephalus bungaroides*, is a small venomous species restricted to a handful of disjunct reserves near Sydney, Australia. Mark-recapture studies have indicated low vagility for this ambush predator, suggesting that gene flow also may be low. However, our analyses of 11 microsatellite loci from 163 snakes collected in Morton National Park, from six sites within a 10-km diameter, suggest relatively high rates of gene flow among sites. Most populations exchange genes with each other, with one large population serving as a source area and smaller populations apparently acting as sinks. About half of the juvenile snakes, for which we could reliably infer parentage, were collected from populations other than those in which we collected their putative parents. As expected from the snakes’ reliance on rocky outcrops during cooler months of the year, most gene flow appears to be along sandstone plateaux rather than across the densely forested valleys that separate plateaux. The unexpectedly high rates of gene flow on a landscape scale are encouraging for future conservation of this endangered taxon. For example, wildlife managers could conserve broad-headed snakes by restoring habitats near extant source populations in areas predicted to be least affected by future climate change.

## Introduction

Many endangered species exist as small fragmented populations. The reasons for population fragmentation sometimes are obvious: for example, in habitat specialists that depend upon discontinuously distributed patches, or in formerly widespread taxa whose range has been fragmented by urbanization and habitat destruction (e.g., Dubey and Shine 2010; Gong et al. 2010; Lee et al. 2010). In other cases, the reasons are unclear, particularly when extant populations are patchily distributed within superficially homogenous landscapes (e.g., Shine et al. 1998). Because restricted gene flow between populations can exacerbate the effects of threatening processes (Frankham et al. 2002; King 2009), understanding the extent of gene flow between populations, and the landscape factors that increase or reduce such genetic connectivity, can help to predict the spatial effects of local perturbations (e.g., Emaresi et al. 2011), identify appropriate units for conservation and management (e.g., Ferchaud et al. 2011), and distinguish between source and sink populations (Dubey et al. 2008, 2009). Quantifying unidirectional migration rates among populations (Beerli and Felsenstein 2001; Wilson and Rannala 2003) can help to define source and sink populations. This latter point is important because the continued viability of sink populations depends upon preventing the destruction or degradation of source habitats and populations (e.g., Tittler et al. 2006).

**Figure 1:**
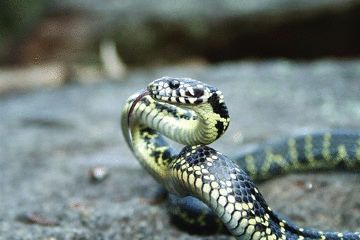
Adult broad-headed snake (*H. bungaroides*) in defensive posture.

Information on the spatial extent and determinants of gene flow is crucial for conserving endangered species that persist as isolated populations. The broad-headed snake (*Hoplocephalus bungaroides*, Elapidae; Fig. 1) is restricted to sandstone rock outcrops within a 200-km radius of Sydney, in south eastern Australia (Webb and Shine 1997a, b). Broad-headed snakes have shown a dramatic historical decline in distribution and abundance, and most extant populations are restricted to national parks (Morton, Yengo, Wollemi, and Blue Mountains NP; Shine et al. 1998). The species has a patchy distribution even in apparently suitable habitat (weathered sandstone rocky outcrops surrounded by woodland; Shine and Fitzgerald 1989; Shine et al. 1998). The processes that threaten the viability of broad-headed snake populations also are spatially heterogeneous. Major threats include the illegal collection of rocks for landscaping urban gardens (Shine et al. 1998), the removal of snakes for the underground pet trade (Webb et al. 2002), and forest thickening that apparently reflects changed fire regimes since European occupation of Australia (Pringle et al. 2009). Such fire effects exhibit strong small-scale spatial heterogeneity and depend on local topography, fuel loads, and wind speed and direction during fire (Bradstock et al. 2002). Vegetation density is a critical determinant of habitat quality for these small snakes, which spend the cooler months of the year in crevices beneath sun-exposed rocks (Webb and Shine 1997a, b, 2008; Shine et al. 1998; Webb et al. 2002a, b).

Mark-recapture studies on this endangered snake species suggest low vagility. Adult snakes often occupy the same rocks in successive winters, and juveniles are rarely recaptured far from their initial capture site (average of 161 m in 6–34 months, Webb and Shine 1997a). By contrast, radio-tagged adult broad-headed snakes moved long distances (up to 750 m) between their winter retreats (rocky outcrops) and their summer habitat (woodland; Webb and Shine 1997a, b). Hence, the deep and densely forested valleys that dissect these ancient sandstone plateaus may not necessarily prevent gene flow among broad-headed snake populations. However, mating in this species likely occurs in the cooler months of the year, when the snakes are concentrated on their sun-exposed rocky ridges (Shine and Fitzgerald 1989). Thus, although adult snakes may move through the forest in summer, they may form isolated breeding populations in rocky sites in winter.

Based on molecular markers (11 microsatellite loci), we conducted parentage and landscape genetics analyses to ask the following questions: (1) do snakes move between plateaux (i.e., through the forested valleys that separate rocky sites), or strictly along plateaux? (2) How much gene flow is there between snake populations separated by long distances of apparently suitable but unoccupied habitat (determined by a lack of samples during surveys) ? and (3) What landscape features affect gene flow among populations of this endangered reptile species?

## Materials and Methods

### Tissue sampling, DNA extraction, and microsatellite analyses

We collected 163 samples of *H. bungaroides* during ecological research conducted from 1998 to 2009, from six locations in Morton National Park in southeastern Australia (see Fig. 1 and Tables 1 and 2 for more details). Total cellular DNA was isolated from small tail clips. Tissue samples were placed in 200 µl of 5% Chelex containing 0.2 mg/mL of proteinase K, incubated overnight at 56°C, and boiled at 100°C for 10 minutes. Eleven microsatellite loci isolated and characterized from *H. bungaroides* (Burns and Houlden 1999; Hb30, Hb48), and other elapid species including *Cryptophis nigrescens* (Stapley et al. 2005; Rn75, Rn114, Rn128), *Notechis scutatus* (Scott et al. 2001; Ns05, Ns14, Ns32, Ns67), and *Aipysurus laevis* (Lukoschek et al. 2005: AL106, AL28) were amplified and scored.

**Table 1:** Number of potential fathers and mothers, and juveniles for the parentage analyses from 1998 to 2009. Adults captured the years before a given year were included as potential parents, provided that such individuals were recaptured either in the year in which hatchlings were born, or in subsequent years

Year	No. of potential fathers	No. of potential mothers	No. of juveniles
1998	7	3	9
1999	6	3	6
2000	8	7	10
2001	7	8	9
2002	11	5	15
2003	6	5	8
2004	10	6	9
2005	6	3	5
2006	7	4	9
2007	4	3	5
2008	4	6	10
2009	6	3	6
Total	82	56	101

**Table 2:** Number of samples, allelic richness (*AR*), expected (*H_S_*) and observed (*H_O_*) heterozygosities, within-subpopulation deviation from random mating (*F*_IS_), as well as mean subpopulation differentiation (*F*_ST_)

Sites	*N*	*AR*	*H_S_*	*H_O_*	*F_IS_*	Mean *F_ST_*
Site1	37	2.70	0.50	0.51	−0.027	0.033
Site2	58	2.69	0.49	0.51	−0.034	0.040
Site3	4	3.00	0.57	0.68	−0.2	0.042
Site4	16	2.71	0.50	0.53	−0.051	0.036
Monkey Gum	36	2.63	0.47	0.43	0.076	0.061
Nerriga	12	2.31	0.44	0.47	−0.06	0.121
Total	163	2.75	0.50	0.52	−0.016	0.044*

* *P* < 0.05.

PCR amplifications were performed in a 9800 Fast thermal cycler (Applied Biosystems, Foster CA, USA) as 5 µl reactions containing 0.075 U *Taq Ti* DNA polymerase (Fisher Biotec, Wembley WA, AU), 0.1-mM dNTPs, 0.4 mM of each primer, 20-mM Tris-HCl, pH 8. 5, 50-mM KCl, 1.25-mM MgCl_2_, and 0.8 µL of DNA extraction. Cycling conditions included a hot start denaturation of 95°C for 3 min, followed by 40 cycles of 95°C for 30 sec, 55°C (58°C for Ns14) annealing temperature for 30 sec, 72°C for 30 sec, and a final extension of 72°C for 7 min. Amplified products were genotyped with a 3130 xl genetic analyzer (Applied Biosystems) using Genemapper software V3.7 (Applied Biosystems).

### Genetic structure and dispersal

To check for linked loci, we calculated the genotypic disequilibrium between loci in each sample based on 10,000 randomizations. Deviations from Hardy–Weinberg equilibrium (HWE) within samples were tested based on 10,000 randomizations. Wright's fixation indices for within-population deviation from random mating (*F_IS_*) were estimated following Weir and Cockerham (1984), and deviations from random mating within populations (*F_IS_*) per locus and sample were computed with a bootstrap procedure (10,000 randomizations). We estimated expected (*H_S_*) and observed (*H_O_*) heterozygosities following the methods of Nei and Chesser (1983). We performed all summary statistics and tests using the software FSTAT Version 2.9.3.2 (Goudet 2002). Significance values were corrected for multiple tests using the sequential Bonferroni method (Rice 1989). We used microchecker (Van Oosterhout et al. 2004) to test for the presence of null alleles.

Mantel and partial Mantel tests (Mantel 1967) were performed using the software FSTAT Version 2.9.3.2 (Goudet 2002), with genetic distance as the dependent variable and one or a combination of the following variables: (1) the distance between sites, (2) the true distance between sites (We used three-dimensional spatial analyst in ArcMap 9.3 to calculate the “true distance” between sites, which incorporated the geographic distance between sites while accounting for topography (elevation; at 25-m resolution); this allowed us to estimate the distance that a snake would have to travel to move between sites), (3) the number of rivers between sites, (4) the difference between the mean elevation of two sites and the minimum elevation of the area between them (reflecting the presence of valleys, and thus, the amount of vertical travel needed to move from one site to the other), and (5) the number of roads (all dirt roads in our dataset) as explanatory variables. *P*-values were calculated after 10,000 randomizations and tests were performed in FSTAT Version 2.9.3.2 (Goudet 2002). Based on these results, we selected the best model using Akaike's information criterion (AIC; Akaike 1973). As our sample size of pairwise comparisons is relatively low (*N*= 15), we converted AIC values into AIC_c_ (Hurvich and Tsai 1989). Then, each candidate model was compared based on its AIC_c_ scores and weights. The best-supported models are those with high Akaike weights and that deviate from the best model by less than two units (i.e., ΔAIC_c_ < 2; Burnham and Anderson 1998).

We estimated migration rate (*m*) using BAYESASS 1.3 (Wilson and Rannala 2003) to estimate short-term gene flow (i.e., during the past one to three generations) between extant *H. bungaroides* populations. This Bayesian method relies on the tendency for immigrants to show temporary disequilibrium in their genotypes relative to the focal population, allowing their identification as immigrants or offspring of immigrants. This software provides unidirectional estimates of *m* for each population pair. Contrary to the other approaches, bayesass does not assume migration-–drift equilibrium, an assumption that is frequently violated in natural populations (Whitlock and McCauley 1999). Initial runs showed that convergence was reached using 3 × 10^6^ Markov Chain Monte Carlo (MCMC) iterations; for the final analysis, we used 3 × 10^7^ MCMC iterations of which 1 × 10^7^ were for the burn-in.

We used the software Migrate 3.2.15 (Beerli and Felsenstein 2001; Beerli 2010) to estimate the population size parameter (θ) and the scaled migration rate (*m*). This software is based on a coalescence model with mutation and migration, and estimates a measure of effective population size, θ, defined as 4*N*_e_µ, where l denotes the mutation rate and *N*_e_ the effective population size, and migration M, defined as *m*?µ, where *m* denotes migration rate. Estimates were based on five long (10^5^ MCMC steps) and 15 short chains (10^4^ MCMC steps). We used, the “adaptive heating" option with one “cold" and three “hot" chains to ensure convergence.

The software Structure 2.1 (Pritchard et al. 2000; Falush et al. 2003) was used to infer population structure and assign individuals to populations. Based on allele frequencies, a MCMC simulation is used to assign each individual a membership coefficient for each of K populations. Ten runs of 1 × 10^5^ iterations (the first 2 × 10^4^ considered as burn-in) for *K*= 1–6, including all the populations were performed. We defined the number of populations best fitting our dataset as described in Evanno et al. (2005). The latter statistic compares the rate of change in the log probability of data between successive *K* and the corresponding variance of log probabilities.

### Parentage analyses

We determined paternity of 101 juvenile snakes on the basis of a maximum likelihood method via the program CERVUS 3.0 (Marshall et al. 1998; Kalinowski et al. 2007), in order to determine the consistency with which offspring are captured in the same populations as their parents, that is, the degree of dispersal. This program conducts parentage testing for parent pairs (when both parents are unknown) and attempts to assign to each offspring the most likely mother, the most likely father, and the most likely parent pair, by calculating an LOD score (i.e., the logarithm of the likelihood ratio) for every potential parent (male and female), as well as for both parents simultaneously (Marshall et al. 1998). The difference between the LOD scores of the parent (male and female, separately) with the highest value and the parent with second highest value is the Δ-criterion (ΔLOD; Marshall et al. 1998). ΔLOD is compared with the critical Δ values calculated after a simulation and provided with a statistical confidence level. The simulations were based on allele frequency data from the adult samples, because sampling of juveniles could skew the analyses if multiple individuals belong to the same litter. Parameters used for the simulation were based on the following criteria (for each year separately): (1) total number of candidate adult males and females; (2) mean proportion of candidate parents sampled; (3) mean proportion of data typed ≥ 0.95 (i.e., ≤ 5% of data missing); and (4) rate of typing errors = 0.05. We chose the confidence level proposed by Marshall et al. (1998; strict confidence ≥ 95% and a relaxed confidence of 80%). In order to minimize incorrect assignments, candidate parents were determined using a size criterion for maturity (SVL > 490 mm, based on the minimum size for males, the smaller sex; Webb et al. 2002) and only parents assigned at ≥ 95% certainty were considered. The estimated proportion of parents sampled was 30% (Webb and Shine 2008).

## Results

### Genetic structure and dispersal

**Figure 2:**
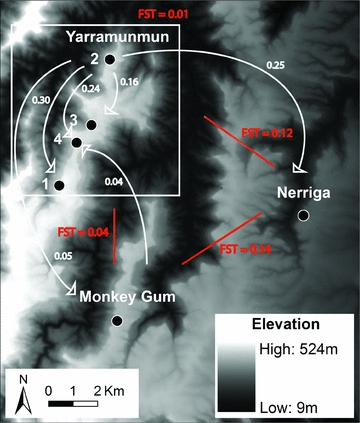
Location of populations of broad-headed snakes, from which tissue samples were obtained, showing elevation (lighter shading shows higher ground; the dark corridors represent streams running through forested valleys between the sandstone plateaus) and pairwise subpopulation differentiation (*F*_ST_) values among the Yarramunmun, Nerriga, and Monkey Gum populations, and major unidirectional migration rates from BAYESASS.

We did not detect any significant linkage disequilibrium or null alleles, or deviation from HWE within our six populations. For the 11 microsatellite loci, the number of alleles per locus ranged from 2 to 22, with a total of 73 alleles across 11 loci. Observed heterozygosity within populations (*H*_O_) varied from 0.43 to 0.68, and expected heterozygosity (*H*_S_ from 0.44 to 0.57 (Table 2). The genetic subdivisions between populations (pairwise *F*_ST_) ranged from –0.0004 to 0.1379 (Fig. 2; Table 3), with an overall *F*_ST_ of 0.044 (*P* < 0.001).

**Table 3:** Pairwise subpopulation differentiation (*F*_ST_; lower matrix) and distance between populations (in meters; upper matrix)

Site 1	Site 2	Site 3	Site 4	Monkey Gum	Nerriga
Site1	3651	1883	941	7449	9707
Site2	0.0131*	5534	2773	10657	10129
Site3	−0.0004	0.0261	2793	6000	10022
Site4	0.0021	0.0077*	0.0127	8052	9391
Monkey Gum	0.0328*	0.0444*	0.0528	0.0362*	8721
Nerriga	0.1155*	0.1094*	0.1209*	0.1220*	0.1379*

* *P* < 0.05.

Based on the Mantel and partial Mantel tests, the number of rivers present between populations was the most important predictor variable for the observed genetic structure (*F*_ST_) in *H. bungaroides* (AIC_c_ value =−51.56; AIC_c_ weight = 0.37; *R*^2^= 93.37; Table 4). The second best model included the number of rivers and roads between sites (AIC_c_ value =–50.98; AIC_c_ weight = 0.27; *R*^2^= 95.55), and the third one, the straight-line distance and the number of rivers between sites (AIC_c_ value =–49.14; AIC_c_ weight = 0.11; *R*^2^= 94.09). There was little difference between the two highest-ranking models (ΔAIC_c_= 0.58). Importantly, all three of the best models include the number of rivers as a significant variable. Because the true distance between sites was no more informative than the straight-line distance, only this latter variable was used in our analyses (see Table 4).

**Table 4:** Results of Mantel and partial Mantel tests performed with genetic distance as the dependent variable and with a listing of variables included in the models (number of rivers [River] and number of roads [Road] between sites, mean elevation of sites minus the minimum elevation between sites [Elevation], straight-line distance [Distance], and true distance between sites [True distance]), the number of parameters per model (K), AICc, **Δ** AICc, AICc weight, and *R*^2^ (total variance explained by the model)

Variable	*K*	AICc	Δ AICc	AICc weight	*R*^2^
River	2	−51.56	−	0.37	93.37
River and road	3	−50.98	0.58	0.27	95.55
Distance and river	3	−49.14	2.42	0.11	94.09
River and elevation	3	−48.38	3.18	0.07	93.37
Distance and road and river	4	−47.84	3.72	0.06	95.99
River and elevation and road	4	−47.23	4.33	0.04	95.59
River and elevation and distance	4	−47.08	4.48	0.04	95.49
Road	2	−44.60	6.96	0.01	80.68
Distance and road	3	−43.91	7.65	0.01	86.82
River and elevation and road and distance	5	−43.82	7.74	0.01	96.37
Road and elevation	3	−42.86	8.7	0.00	84.51
Distance	2	−40.94	10.62	0.00	66.10
True distance	2	−40.92	10.64	0.00	66.02
Distance and road and elevation	4	−40.17	11.39	0.00	86.98
Distance and elevation	3	−39.43	12.13	0.00	73.80
Elevation	2	−36.12	15.44	0.00	29.01

The BAYESASS analysis suggested a recent exchange of migrants between most of the populations, but in an asymmetric fashion (i.e., standard deviations did not overlap; Table 5; Fig. 2) with a maximum *m* of 0.30 (std: 0.02) from Site 2 into Site 1 (distance: 3,651m). Site 2 appeared to be involved in most of the recent gene flow between populations. Similarly, the Migrate analysis suggested that migration occurs among the populations (Table 6), with values of M ranging from 0.33 to 2.84.

**Table 5:** Unidirectional migration-rate estimates within pairs of *H. bungaroides* populations from BAYESASS (*m*; Wilson and Rannala 2003). The populations into which individuals are migrating are listed in the rows, while the origins of the migrants are listed in the columns. Migrations rates ≥ 0.10 are in italics. Estimates are followed by the standard deviation in parentheses

Pop	Site 1	Site 2	Site 3	Site 4	Monkey Gum	Nerriga
Site 1	——	0.00 (0.01)	0.03(0.04)	0.02 (0.02)	0.00 (0.01)	0.02 (0.06)
Site 2	*0.30* (0.02)	——	*0.16* (0.07)	*0.24* (0.04)	0.05 (0.04)	*0.25* (0.06)
Site 3	0.00 (0.01)	0.00 (0.01)	——	0.01 (0.01)	0.01 (0.04)	0.01 (0.02)
Site 4	0.01 (0.01)	0.00 (0.00)	0.03 (0.03)	——	0.00 (0.01)	0.01 (0.02)
Monkey Gum	0.01 (0.01)	0.01 (0.02)	0.03 (0.03)	0.04 (0.03)	——	0.01 (0.02)
Nerriga	0.00 (0.01)	0.00 (0.01)	0.03 (0.03)	0.01 (0.01)	0.00 (0.01)	——

**Table 6:** Scaled estimates of migration rate (*M*= m/µ) between pairs of *H. bungaroides* populations and population size parameter (θ) from Migrate (Beerli 2010). The 95% confidence intervals are given in parentheses

Pop	θ	Site 1	Site 2	Site 3	Site 4	Monkey Gum	Nerriga
Site 1	0.98	/	2.02	1.12	2.64	1.55	2.37
	(0.91–1.06)		(1.65–2.44)	(0.79–1.49)	(1.90–3.22)	(0.90–1.94)	(1.97–2.95)
Site 2	0.94	1.27	/	1.95	2.84	1.51	1.47
	(0.88–1.04)	(0.99–1.60)		(1.60–2.35)	(2.20–3.32)	(1.20–1.87)	(1.17–1.98)
Site 3	0.86	1.36	1.27	/	1.51	1.47	0.33
	(0.68–1.00)	(1.07–1.70)	(0.99–1.60)		(1.09–1.86)	(1.09–1.81)	(0.20–0.55)
Site 4	0.94	1.48	0.87	1.62	/	1.70	1.14
	(0.81–1.09)	(1.20–1.79)	(0.53–1.36)	(1.19–2.02)		(1.40–2.27)	(0.91–1.41)
Monkey Gum	1.03	0.64	0.77	1.07	1.59	/	1.12
	(0.93–1.14)	(0.42–0.82)	(0.59–0.97)	(0.62–1.33)	(1.07–1.88)		(0.83–1.38)
Nerriga	0.80	1.13	0.80	0.85	0.56	1.35	/
	(0.69–0.89)	(0.80–1.42)	(0.45–1.04)	(0.65–1.10)	(0.40–0.76)	(1.04–1.65)	

**Figure 3:**
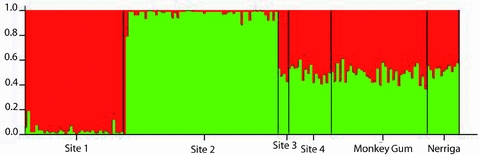
Summary plot of the individual assignment results of the Structure analyses for *K*= 2.

The program Structure revealed two clusters within our dataset, using the method of Evanno et al. (2005). Site 1 belonged to the first cluster, and site 3 to the second cluster, whereas site 3, 4, Nerriga, and Monkey Gum showed a proportion of membership in each of the clusters, close to 50%. For a summary plot, see Fig. 3

### Parentage analyses

Of the 101 juveniles tested, 13 (12.9%) and 48 offspring (47.5%) were assigned at ≥ 95% and ≥80% certainty, respectively, to at least one parent (total of 61). Four offspring had both parents assigned at ≥80%, and another five had one of the parents assigned at ≥95% and the other at ≥80%. Only five trios (parents plus offspring) were assigned at ≥80%, and none at ≥95%. Seven of 13 offspring assigned at ≥ 95% to a parent were collected in the same population as their putative parents, whereas the other six were allocated to parents collected in a different population from their own (Table 7). The maximum distance between collection sites for a juvenile and its presumed parent was about 10,700 m (Site 2—Monkey Gum), and the overall mean distance between pairs of parent plus offspring was 2,940 m. The mean distances between the collection site of a juvenile snake and that of its putative mother and father were 3,540 and 924 m, respectively.

**Table 7:** Juvenile broad-headed snakes for which we were able to assign parentage at ≥95% certainty, showing snake identification numbers and sites of collection

Year	Offspring ID	Offspring site	Mother ID	Mother site	Father ID	Father site
1998	Hb045	Site 2	Hb056	Site 2	/	/
1998	Hb110	Site 1	Hb061	Site 2	/	/
1999	Hb100	Site 1	Hb050	Site 1	/	/
1999	Hb114	Site 1	Hb061	Site 2	/	/
2001	Hb039	Nerriga	/	/	Hb033	Nerriga
2002	Hb080	Site 1	Hb093	Site 1	/	/
2003	Hb187	Site 1	/	/	Hb071	Site 1
2004	Hb139	Monkey Gum	HB142	Monkey Gum		
2004	Hb147	Monkey Gum	Hb169	Nerriga	/	/
2005	DAP0214	Monkey Gum	Hb045	Site 2	/	/
2005	Hb215	Site 4	/	/	Hb008	Site 2
2006	Hb190	Site 2	Hb045	Site 2	/	/
2007	DAP0205	Monkey Gum	HB169	Nerriga	/	/

## Discussion

Our genetic analyses show that broad-headed snakes are not as sedentary as mark-recapture studies have suggested (e.g., Webb and Shine 1997a). Indeed, our results indicate significant gene flow over relatively large spatial scales (maximum distance between sites: 10.7 km; mean overall *F*_ST_= 0.04), with a maximum distance between collection sites of an offspring and its putative parent of about 10,700 m (based on parentage analyses). Strikingly, almost half (46%) of the juvenile snakes assigned at ≥95% appear to be the progeny of parents that were collected from sites other than those in which the juveniles were collected, consistent with the overall low structure found between populations. As predicted from the snakes’ reliance upon rocky habitats in winter, most dispersal occurred between outcrops on the same plateaux rather than across valleys separating adjacent plateaux (see Fig. 1). In addition, our landscape genetic analyses identified rivers as the major barrier to gene flow between populations. Most of the rivers in this area are small, and broad-headed snakes could easily cross them (personal observation). Thus, it appears that rivers interrupt gene flow in this species not because they pose a physical barrier to snake dispersal, but because snakes rarely use riparian habitats in our study area (Webb and Shine 1997a, b).

Gene flow in *H. bungaroides* is slightly lower than observed in widespread Australian snake species such as the small-eyed snake *C. nigrescens* (mean *F*_ST_: 0.02; distance between populations: 0.7–1.9 km; Keogh et al. 2007) or the slatey-grey snake (*Stegonotus cucullatus*; mean *F*_ST_: 0.012; distance between populations: 3–6 km; Dubey et al. 2008). However, unidirectional migration rates (*m*; including the past one to three generations; Wilson and Rannala 2003) were comparable to this latter species, capable of long-distance dispersal, with rates of up to 0.3 (Dubey et al. 2008). Wilson and Rannala (2003) considered migration rates (*m*) between populations of more than 0.20 as important. Interestingly, the gene flow is much higher than in endangered species, like the Orsini's viper *Vipera u. ursinii* in France (Ferchaud et al. 2011), the adder *V. berus* in Switzerland (mean *F*_ST_: 0.27; Ursenbacher et al. 2009), or the eastern massasauga rattlesnake *Sistrurus c. catenatus* in North America (Chiucchi and Gibbs 2010). Nevertheless, these differences could be attributed to the presently highly fragmented habitat of these three species, whereas our *H. bungaroides* populations are distributed in a national park. Similarly, a lack of genetic structure in the specialized grass snakes *Natrix natrix* from western Switzerland, despite a highly fragmented habitat, has been attributed to interconnection of suitable habitat patches (Meister et al. 2010).

A previous analysis of genetic structure at a larger spatial scale in this species, based on molecular phylogeny (Sumner et al. 2010), revealed an ancient (mid-Pleistocene) split between northern and southern populations. Interestingly, its sister species *H. stephensii* shows a similar pattern, with northern and southern populations isolated from each other at about the same period. However, *H. stephensii* showed very little substructuring of populations across most of its geographic range, presumably reflecting the availability of continuously forested habitat for this arboreal species until relatively recent times (Keogh et al. 2003). The habitat features upon which *H. bungaroides* depends (sun-exposed sandstone rock outcrops) are more specific, and more fragmented in their distribution, than are the forests used by *H. stephensii*. That difference in spatial heterogeneity of critical habitat features over long (evolutionary) time periods may explain why we see more population structure within the saxicolous *H. bungaroides* than the forest-dwelling *H. stephensii*.

The dispersal distances of *H. bungaroides* inferred from genetic data are higher than earlier estimates based on intensive mark-recapture studies. Genetic data can reveal even low rates of long-distance migration among populations, whereas mark-recapture studies are unlikely to reveal such occasional events (e.g., Hamill et al. 2007; Dubey et al. 2008). For example, a 19-year mark-recapture study of broad-headed snakes (>250 individuals, >350 captures) has only detected one snake (an adult male) migrating between different study sites (Webb, unpubl. data). During a 3-year mark-recapture study, Webb and Shine (1997a) reported a maximum dispersal distance of juvenile broad-headed snakes of 375 m in 6 months. By contrast, our paternity analyses suggest that juveniles are capable of long-distance dispersal. Three of the five juveniles that migrated from their natal sites (Table 7) were 3-year-old snakes, suggesting that dispersal occurs during the first few years of life. However, adults also move long distances (up to 780 m) from winter rock outcrops to the summer woodland habitat (Webb and Shine 1997a). Some marked adult male snakes have vanished for years from sites and then reappeared in the same outcrop as they were originally found, suggesting long-distance displacement followed by return (Webb, unpubl. data). Thus, we cannot identify the life-history stage responsible for the dispersal indicated by our analyses of gene flow. Adults of the sister species *H. stephensii* are highly vagile, moving up to 244 m in a single night (Fitzgerald et al. 2002). The two species share many morphological and behavioral similarities, and are genetically very close (Keogh et al. 2003), suggesting that *H. bungaroides* is capable of rapid long-distance dispersal. Our study reinforces the usefulness of combining field data and genetic analyses to clarify dispersal of *H. bungaroides*, a combination that has revealed landscape-scale movements and mating systems in other secretive reptile species (e.g., Keogh et al. 2007; Dubey et al. 2008).

Our data have several implications for the management of the endangered broad-headed snake. First, given the relatively low genetic structure and important recent gene flow observed between sites, these populations are likely a metapopulation rather than a set of genetically distinct units. However, four of the six populations that we studied appear to be sinks (based on unidirectional gene flow), suggesting that managers should give high priority to protecting the source populations, as well as to improving the habitat quality of sink populations. Encouragingly, the high mobility of this snake species means that local extirpation will not necessarily result in a long-term absence of the species from the affected site, because such areas are likely to be subsequently recolonized from nearby areas (as long as populations in those source areas remain viable). That ability to recolonize may well prove to be crucial for the continued persistence of broad-headed snakes, because climate-change models have predicted range reductions of up to 86% by 2070, and an increasingly patchy distribution of the remaining suitable habitat (Penman et al. 2010). The local scale of currently recognized threatening processes (rock and snake theft, fires, vegetation encroachment; Webb and Shine 1997a,b; Webb et al. 2002a,b;) ultimately may pose different threats than the landscape scale changes likely to be wrought by climate change. Consequently, habitat restoration of sites near extant populations of broad-headed snakes could help to ensure the long-term survival of the species. Recent experimental work has demonstrated the feasibility of such habitat restoration, via selective removal of trees that shade rock outcrops (Pike et al. 2011a,b;) and the deployment of artificial rocks that provide suitable crevices for broad-headed snakes and their prey (Croak et al. 2010). Translocation of individuals to maintain genetic variation within populations (as has been used to reverse declines in severely inbred snake populations: Madsen et al. 1995, 1999) is unlikely to be necessary for broad-headed snakes, given their ability to exchange genes among adjacent populations. Finally, we need to maintain broadly contiguous areas that offer the combination of summer (woodland) and winter (sandstone outcrop) habitat needed for *H. bungaroides*, if this endangered species is to persist in the face of the multiple threatening processes that have caused its historical decline.
